# Burnout and Its Relationship With Depressive Symptoms in Medical Staff During the COVID-19 Epidemic in China

**DOI:** 10.3389/fpsyg.2021.616369

**Published:** 2021-03-04

**Authors:** Lijuan Huo, Yongjie Zhou, Shen Li, Yuping Ning, Lingyun Zeng, Zhengkui Liu, Wei Qian, Jiezhi Yang, Xin Zhou, Tiebang Liu, Xiang Yang Zhang

**Affiliations:** ^1^Department of Psychiatry, Affiliated Brain Hospital of Guangzhou Medical University (Guangzhou Huiai Hospital), Guangzhou, China; ^2^Department of Psychiatric Rehabilitation, Shenzhen Kangning Hospital, Shenzhen, Guangdong, China; ^3^Department of Psychiatry, College of Basic Medical Sciences, Tianjin Medical University, Tianjin, China; ^4^The First School of Clinical Medicine, Southern Medical University, Guangzhou, China; ^5^CAS Key Laboratory of Mental Health, Institute of Psychology, Chinese Academy of Sciences, Beijing, China; ^6^Shenzhen Health Development Research Center, Shenzhen, China; ^7^Research Center for Psychological and Health Sciences, China University of Geosciences, Wuhan, China

**Keywords:** prevalence, medical staff, COVID-19, depression, burnout

## Abstract

**Objective:**

The large-scale epidemic of Coronavirus Disease 2019 (COVID-19) has triggered unprecedented physical and psychological stress on health professionals. This study aimed to investigate the prevalence and risk factors of burnout syndrome, and the relationship between burnout and depressive symptoms among frontline medical staff during the COVID-19 epidemic in China.

**Methods:**

A total of 606 frontline medical staff were recruited from 133 cities in China using a cross-sectional survey. The Maslach Burnout Inventory (MBI) was used to assess the level of burnout. Depressive symptoms were assessed by the Patient Health Questionnaire Depression (PHQ-9).

**Results:**

During the COVID-19 pandemic, 36.5% of the medical staff experienced burnout. Personal and work-related factors were independently associated with burnout, including age (OR = 0.68, 95% CI: 0.52–0.89, *p* = 0.004), family income (OR = 0.72, 95% CI: 0.53–0.99, *p* = 0.045), having physical diseases (OR = 2.16, 95% CI: 1.42–3.28, *p* < 0.001), daily working hours (OR = 1.35, 95% CI: 1.03–1.77, *p* = 0.033), and profession of nurse (OR = 2.14, 95% CI: 1.12–4.10, *p* = 0.022). The correlation coefficients between the scores of each burnout subscale and the scores of depressive symptoms were 0.57 for emotional exhaustion, 0.37 for cynicism, and −0.41 for professional efficacy (all *p* < 0.001).

**Conclusions:**

Our findings suggest that the prevalence rate of burnout is extremely high among medical staff during the COVID-19 pandemic, which is associated with other psychological disorders, such as depression. Psychological intervention for medical staff is urgently needed. Young and less experienced medical staff, especially nurses, should receive more attention when providing psychological assistance.

## Introduction

The outbreak of coronavirus disease 2019 (COVID-19) first appeared in Wuhan, China in December 2019, and has since swept the world at an incredible speed. As of December 2020, there have been more than 70 million confirmed cases and more than 1.7 million deaths^1^. Due to high contagion and possible asymptomatic transmission, as well as a lack of knowledge of the virus, the demand and pressure on frontline medical staff have increased dramatically, especially in the early stages of the pandemic (Hu B. et al., 2020; Li et al., 2020). This condition has seriously aggravated the mental fatigue of healthcare professionals.

According to the latest World Health Organization’s International Disease Classification (ICD-11), burnout is officially classified as an occupational health syndrome, which is characterized by emotional and mental exhaustion due to long-term workplace stress and negative job perception. The most recognized definition of burnout is the three-dimensional psychological syndrome proposed by Maslach and Jackson (1981) that includes emotional exhaustion, cynicism, and reduced professional efficacy. Medical staff are susceptible to job burnout, which has attracted more and more attention recently (Dzau et al., 2018). Meta-analyses have shown that the pooled prevalence of burnout among medical staff is estimated to be about or more than 30% (Dimou et al., 2016; Gómez-Urquiza et al., 2017; O’Connor et al., 2018; Rezaei et al., 2018), a rate of more than twice compared with professionals in other fields (Dzau et al., 2018). Because of the nature of their work, medical staff often face a lot of pressure and negative emotions, such as heavy workload, poor doctor-patient relationship (especially in mainland China), and accumulated frustration in the face of death (He and Qian, 2016; Alharbi et al., 2019). Job burnout reduces working efficiency and increases medical errors (Patel et al., 2018; Tawfik et al., 2019). To make matters worse, burnout may lead to other severe mental disorders, including alcohol abuse/dependence, depression, and an increased risk of suicide (Dyrbye et al., 2008; Johnson et al., 2018).

The unprecedented outbreak of COVID-19 has further damaged the mental health of health care workers. During this pandemic, many social and environmental factors lead to burnout of medical staff, such as isolation, expanded workloads, life-threatening workplaces, concern about infecting relatives or colleagues, and some personal factors (Lai et al., 2020). A number of insightful commentaries have been published to appeal to the mental burden of medical staff, and to propose guidelines and expert consensus on mental health services (Greenberg et al., 2020; Liu et al., 2020; Raudenská et al., 2020). Many surveys have also reported that health care workers exposed to COVID-19 suffered from serious psychological disturbances, the most common of which were depression, anxiety, insomnia, and fear (Lai et al., 2020; Que et al., 2020; Tan et al., 2020a; Zhang W. R. et al., 2020). However, so far, few quantitative studies have investigated the symptoms of job burnout among medical staff (Hu D. et al., 2020; Matsuo et al., 2020; Tan et al., 2020b; Zhang Y. et al., 2020). These studies evaluated burnout symptoms, and focused on frontline nurses instead of estimating prevalence (Hu D. et al., 2020; Zhang Y. et al., 2020), or collecting information in one single institution (Matsuo et al., 2020; Zhang Y. et al., 2020). Tan et al. (2020b) started the survey half a year after the outbreak of the pandemic in China and 4 months after the outbreak in Singapore. At that time, the pressure of medical staff was different (Tan et al., 2020b). Further, they did not separately analyze the three recognized dimensions of burnout due to the use of other tools.

The purposes of this study were: (1) to explore the prevalence of burnout in the frontline medical staff in China during the early stage of COVID-19 epidemic; (2) to identify the individual and job-related determinants of burnout in this population, and (3) to determine the relationship between burnout and depressive symptoms.

## Materials and Methods

### Study Design and Participants

This was a cross-sectional survey designed to assess the job burnout and other mental conditions of frontline medical workers in China during the COVID-19 epidemic. In order to avoid face-to-face interaction, an online questionnaire was constructed and distributed via WeChat, one of the most important social tools in mainland China. Data were collected from February 14 to March 29, 2020. A total of 606 frontline medical workers were recruited from 133 cities across the country. Doctors, nurses, or medical technicians in hospitals, aged 18 years or above were included in this study.

The study was approved by the Institute of Psychology, Chinese Academy of Sciences. Each participant signed an electronic informed consent form before the survey. The information of all respondents was confidential.

### Assessments for Burnout and Depressive Symptoms

Demographic and work-related information was collected, including residence, age, sex, height, weight, ethnicity, marital status, education, annual family income, occupation, department, length of service, and daily working hours. At the same time whether relatives or friends were infected, financial loss, and whether they had experienced SARS outbreaks were also collected.

The Chinese version of the Maslach Burnout Inventory-General Survey (MBI-GS)(Maslach and Jackson, 1981; Schutte et al., 2000) was used to assess job burnout, which has been widely used among healthcare workers in China, and has satisfactory reliability and validity (Wu et al., 2007). The MBI-GS consists of 15 items, measuring three dimensions of occupational burnout: emotional exhaustion (EE), which means being emotionally depleted at work; cynicism (CY), which means negative or cynical attitudes toward work; professional efficacy (PE), which means a positive sense of success/achievement at work. Each item is scored using a 7-point frequency range scale (0 = never to 6 = daily). The total score of each subscale is stratified into high, moderate, or low tertiles. Based on the previous large sample studies on Chinese healthcare workers (Wu et al., 2014), the cutoffs for each tertile of burnout were determined as follows: low EE < 9, moderate EE 9–13, high EE > 13; low CY < 3, moderate CY 3–9, high CY > 9; low PE > 30, moderate PE 30–18, high PE < 18. A score in the highest tertiles of EE, in combination with the highest tertiles of CY or the lowest tertiles of PE indicates burnout syndrome, according to the “exhaustion + 1” criterion (Brenninkmeijer and VanYperen, 2003). Since the definition of burnout varies considerably in the literature, the prevalence of burnout was also calculated using an alternative formula, a more restrictive definition, that is, a combination of a high EE and high CY and low PE subscale score (Lin et al., 2019). Patient Health Questionnaire-9 (PHQ-9) was applied to assess depressive symptoms (Kroenke et al., 2001). PHQ-9 consists of 9 items, each with a score from 0 to 3. People with a total score of 4 or more are identified to have depressive symptoms.

### Statistical Analysis

The chi-square test and analysis of variance (ANOVA) were used to compare the demographic and work-related variables of participants between the burnout group and the non-burnout group. The binary logistic regression model was used to find out factors independently related to burnout experience. Then, in order to further identify the independent factors associated with MBI-GS scores, stepwise multivariate linear regression models were used, with the MBI-GS subscores as dependent variables, and other variables with potential correlation (*p* < 0.1) with MBI-GS scores as independent factors. Associations between MBI-GS subscale scores and PHQ-9 scores were examined using Pearson correlation analysis and then linear regression model. Bonferroni corrections were applied to adjust multiple tests. A two-tailed test at *p* < 0.05 was set to be statistically significant. All statistical analyses were conducted using SPSS (version 24.0).

## Results

### Demographic Characteristics

Among all the participants, 492 (81.2%) were female and 114 (18.8%) were male. The age of participants ranged from 22 to 65 years old, with an average age of 35.77 ± 8.13 years. The average BMI was 23.34 ± 5.61 Kg/m^2^. More detailed information about the demographic and job-related characteristics of participants is shown in Table 1.

**TABLE 1:** Demographic data of participants with and without burnout.

Variable	Total (*n* = 606)	Non-burnout (*n* = 385)	Burnout (*n* = 221)	*p*-value
**Age**				<0.001
<30	177 (29.2%)	102 (57.6%)	75 (42.4%)	
30–40	261 (43.1%)	155 (59.4%)	106 (40.6%)	
>40	168 (27.7%)	128 (76.2%)	40 (23.8%)	
**Sex**				0.012
Male/Female (male%)	114/492 (18.8%)	84/301 (21.8%)	30/191 (13.6%)	
**BMI**				0.682
<18.5	43 (7.1%)	29 (67.4%)	14 (32.6%)	
18.5–24	379 (62.5%)	243 (64.1%)	136 (35.9%)	
>24	183 (30.2%)	112 (61.2%)	71 (38.8%)	
**Education**				0.488
High school degree or lower, n (%)	14 (2.3%)	7 (50%)	7 (50%)	
College degree, n (%)	446 (73.6%)	282 (63.2%)	164 (36.8%)	
Master or Doctoral degree, n (%)	146 (24.1%)	96 (65.8%)	50 (34.2%)	
**Marital status**				0.455
Single, n (%)	123 (20.6%)	73 (59.3%)	50 (40.7%)	
Married, n (%)	456 (74.9%)	293 (64.3%)	163 (35.7%)	
Widowed or divorced	27 (4.5%)	19 (70.4%)	8 (29.6%)	
**Ethnicity**				0.493
Han/Non-han population (Han%)	556/50 (91.7%)	351/34 (91.2%)	205/16 (92.8%)	
**Family income**				0.018
Low	106 (17.5%)	56 (52.8%)	50 (47.2%)	
Medium	402 (66.3%)	259 (64.4%)	143 (35.6%)	
High	98 (16.2%)	70 (71.4%)	28 (28.6%)	
**Physical diseases**				0.003
Yes/No (Yes%)	133/473 (21.9%)	70/315 (18.2%)	63/158 (28.5%)	
**Infected relatives or friends**				0.058
Yes/No (Yes%)	13/593 (2.1%)	5/380 (1.3%)	8/213 (3.6%)	
**Experienced SARS**				0.199
Yes/No (Yes%)	262/344 (43.2%)	174/211 (45.2%)	88/133 (39.8%)	
**Profession**				0.001
Doctor	208 (34.3%)	143 (68.8%)	65 (31.3%)	
Nurse	334 (55.1%)	192 (57.5%)	142 (42.5%)	
Medical technician	64 (10.6%)	50 (78.1%)	14 (21.9%)	
**Length of service**				0.036
<6 years	110 (18.2%)	69 (62.7%)	41 (37.3%)	
6–10 years	163 (26.9%)	92 (56.4%)	71 (43.6%)	
11–20 years	194 (32%)	123 (63.4%)	71 (36.6%)	
>20 years	139 (22.9%)	101 (72.7%)	38 (27.3%)	
**Daily working hours**				0.190
4–8	284 (46.9%)	190 (66.9%)	94 (33.1%)	
8–10	268 (44.2%)	165 (61.6%)	103 (38.4%)	
> 10	54 (8.9%)	30 (55.6%)	24 (44.4%)	

### Prevalence of Burnout in Medical Staffs

Burnout was defined as a high EE combing with a high CY or low PE subscale scores. During the epidemic of COVID-19, 36.5% of medical staff met the criteria for burnout in our sample. The prevalence of burnout in female workers was significantly higher than that in male workers, whether it was the inclusive criteria (38.8% vs. 26.3%, χ^2^ = 6.25, *p* = 0.012) or the restrictive one (30.5% vs. 16.7%, χ^2^ = 8.79, *p* = 0.003). For each component of burnout, the prevalence of EE, CY, and PE was 40.9, 63.7, and 46%, respectively. In addition, under the strictest definition, combining the highest level of EE and CY and the lowest level of PE, the overall prevalence of burnout was 27.8%.

Chi-squared tests also revealed that there were significant differences between burnout and non-burnout groups in terms of age, annual family income, physical disease, occupation, and service time (all *p* < 0.05). The burnout rates of each type of variables were shown in Table 1. Specifically, medical staff with younger age, female gender, lower family income, more severe physical disease, shorter service, and nursing profession had more severe syndrome of burnout. Individuals having relatives or friends infected with COVID-19 were at a marginally higher risk experiencing burnout (*p* = 0.058). There was no significant difference in BMI, education, marital status, ethnicity, experienced SARS or not, and daily working hours (all *p* > 0.05) between the burnout and non-burnout groups.

Further, the binary logistic regression model revealed that the following variables were independently associated with burnout, including age (OR = 0.68, 95% CI: 0.52–0.89, *p* = 0.004), family income (OR = 0.72, 95% CI: 0.53–0.99, *p* = 0.045), having physical disease (OR = 2.16, 95% CI: 1.42–3.28, *p* < 0.001), daily working hours (OR = 1.35, 95% CI: 1.03–1.77, *p* = 0.033), and profession of nurse (OR = 2.14, 95% CI: 1.12–4.10, *p* = 0.022).

### Factors Associated With Burnout and Its Three Components in Medical Staffs

The average burnout score was 11.94 ± 6.47 on EE subscale, 10.27 ± 4.74 on CY subscale, and 19.25 ± 6.72 on PE subscale. MBI-GS subscale scores after grouping according to demographics and work-related variables were present in Table 2. Then multiple linear regressions were performed to identify independent related factors to each MBI-GS subscore. EE was independently correlated with age (β = −0.13, *t* = −2.87, *p* = 0.004), physical diseases (β = 0.12, *t* = 3.0, *p* = 0.003), professional role of nurses (β = 0.09, *t* = 2.16, *p* = 0.031), and daily working hours (β = 0.14, *t* = 3.57, *p* < 0.001). CY was independently correlated with professional role of nurses (β = 0.21, *t* = 4.90, *p* < 0.001), age (β = −0.11, *t* = −2.45, *p* = 0.015), and family income (β = −0.10, *t* = −2.40, *p* = 0.017). PE was independently correlated with age (β = 0.13, *t* = 3.06, *p* = 0.002) and professional role of nurses (β = −0.30, *t* = −7.18, *p* < 0.001). Taken together, younger age and nursing profession were independently correlated with all dimensions of burnout.

**TABLE 2:** MBI-GS subscale scores in grouped demographics and work-related variables.

Variables	EE	CY	PE
**Age, years**			
<30	12.69 ± 6.70	11.11 ± 4.53	17.77 ± 6.15
30–40	12.41 ± 6.45	10.85 ± 4.44	18.32 ± 6.24
>40	10.43 ± 6.05	8.47 ± 4.96	22.23 ± 7.10
*F*	6.54**	17.73**	25.18**
**Sex**			
Male	10.79 ± 5.92	8.84 ± 4.70	21.54 ± 6.8
Female	12.21 ± 6.57	10.60 ± 4.70	18.72 ± 6.59
*F*	4.49*	12.89**	16.73**
**BMI**			
<18.5	11.16 ± 6.03	11.16 ± 4.35	18.05 ± 6.53
18.5–24	11.90 ± 6.5	10.47 ± 4.62	18.99 ± 6.64
>24	12.24 ± 6.54	9.61 ± 5.03	20.05 ± 6.88
*F*	0.51	2.87	2.26
**Education**			
High school degree or lower	12.71 ± 7.69	11.71 ± 1.90	17.93 ± 3.56
College degree	11.93 ± 6.58	10.71 ± 4.69	18.57 ± 6.60
Master or Doctoral degree	11.91 ± 6.06	8.77 ± 4.79	21.45 ± 6.86
*F*	0.10	10.11**	10.70**
**Marital status**			
Single	12.55 ± 6.60	10.71 ± 4.84	18.31 ± 6.74
Married	11.78 ± 6.49	10.16 ± 4.71	19.43 ± 6.69
Widowed or divorced	11.85 ± 5.70	9.96 ± 4.94	20.44 ± 6.90
*F*	0.67	0.69	1.80
**Ethnicity**			
Han population	11.93 ± 6.42	10.36 ± 4.71	19.11 ± 6.73
Non-han population	12.10 ± 7.11	9.18 ± 5.08	20.74 ± 6.48
*F*	0.32	2.86	2.70
**Family income**			
Low	13.17 ± 6.89	11.42 ± 3.97	18.06 ± 5.62
Medium	11.79 ± 6.35	10.38 ± 4.79	18.99 ± 6.73
High	11.27 ± 6.41	8.55 ± 4.91	21.58 ± 7.25
*F*	2.57	9.91**	8.06**
**Physical diseases**			
No	11.60 ± 6.46	10.22 ± 4.71	19.26 ± 6.70
Yes	13.18 ± 6.40	10.41 ± 4.89	19.21 ± 6.81
*F*	6.27*	0.17	0.01
**Infected relatives or friends**			
No	11.90 ± 6.48	10.25 ± 4.72	19.24 ± 6.68
Yes	14.08 ± 6.06	10.77 ± 5.93	19.38 ± 8.72
*F*	1.44	0.15	0.01
**Experienced SARS**			
No	11.92 ± 6.59	10.52 ± 4.63	18.83 ± 6.79
Yes	11.98 ± 6.33	9.94 ± 4.88	19.79 ± 6.60
*F*	0.2	2.25	3.08
**Profession**			
Doctor	11.38 ± 6.15	8.54 ± 4.94	22 ± 6.85
Nurse	12.69 ± 6.65	11.43 ± 4.22	17.13 ± 5.89
Medical technician	9.89 ± 6.03	9.77 ± 4.96	21.33 ± 6.53
*F*	6.36**	26.21**	42.14**
**Length of service**			
<6 years	12.06 ± 7.05	10 ± 5.13	19.47 ± 7.22
6–10 years	12.96 ± 6.31	11.61 ± 4.20	17.14 ± 5.30
11–20 years	11.81 ± 6.22	10.36 ± 4.88	19.28 ± 6.67
>20 years	10.84 ± 6.41	8.78 ± 4.96	21.50 ± 7.14
*F*	2.75*	9.42**	11.14**
**Daily working hours**			
4–8	11.13 ± 6.24	10.43 ± 4.58	19.18 ± 6.56
8–10	12.48 ± 6.43	10.37 ± 4.68	19.09 ± 6.71
>10	13.57 ± 7.38	8.89 ± 5.67	20.39 ± 7.59
*F*	4.93**	2.52	0.87

*p < 0.05, **p < 0.01. MBI-GS, Maslach Burnout Inventory-General Survey; EE, emotional exhaustion; CY, cynicism; PE, professional efficacy.

### The Association Between Burnout and Depressive Symptoms in Medical Staffs

**FIGURE 1:**
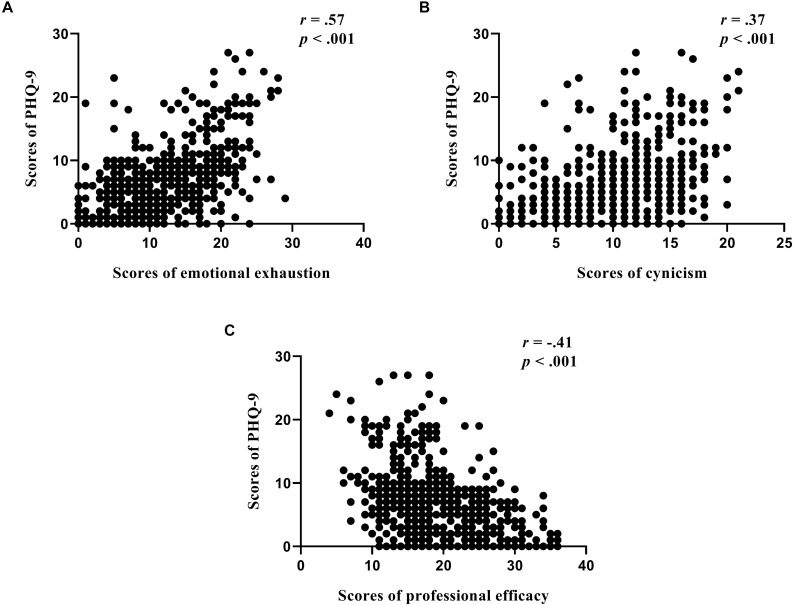
Three dimensions of burnout **(A)** emotional exhaustion, **(B)** cynicism, and **(C)** professional efficacy were associated with depressive symptoms (PHQ-9) in medical staff.

The mean score of PHQ-9 was 6.46 ± 5.57. With the cut-off score of 4, the overall prevalence of depressive symptoms in medical staff was 57.6%. The correlation coefficients between the score of each MBI-GS subscale and the score of PHQ-9 were 0.57 for EE, 0.37 for CY, and −0.41 for PE (all *p* < 0.001, Figure 1). These associations remained significant after Bonferroni corrections. Stepwise multiple regression model showed that scores of EE (β = 0.51, *t* = 12.12) and PE (β = 0.51, *t* = 12.12) were independently associated with PHQ-9 score. These two components of burnout together accounted for 32.8% of the variance (adjusted R^2^) in PHQ-9 (*F* = 148.75, *p* < 0.001).

## Discussion

Although there has been a large number of studies on mental health problems caused by the COVID-19 pandemic, only a few have investigated burnout syndrome, which was particularly prevalent in medical staff even before this pandemic. To our knowledge, this is the first nationwide cross-sectional survey on job burnout of medical staff during the COVID-19 pandemic, with a total of 606 participants. The main findings of this study were: (1) up to 36.5% of the respondents met the criteria of burnout; (2) personal factors (i.e., age, sex, physical diseases, and family income) and job-related factors (daily working hours, length of service, and nursing profession) were associated with burnout; (3) the burnout levels were associated with the severity of depressive symptoms.

Our results showed an extremely high prevalence (36.5%) of burnout, which made medical workers psychologically vulnerable in this pandemic. Consistently, previous studies have revealed that medical workers are particularly prone to burnout. The prevalence of burnout in healthcare professions varies from 12.6 to 76.9% in different studies (Abdulla et al., 2014; Adriaenssens et al., 2015; Elmore et al., 2016; Wen et al., 2016; Gómez-Urquiza et al., 2017). The huge differences across studies not only result from regional disparities but also result from different approaches to define burnout (Rotenstein et al., 2018). It is still in dispute whether the concept of burnout should be regarded as a unidimensional or multidimensional construct. Some research defined burnout as a multidimensional construct, so individuals were considered burnout when meeting the criterion of one of the three MBI dimensions (Elmore et al., 2016; Gómez-Urquiza et al., 2017). While other studies combine different dimensions into a unidimensional burnout, which also develop many formulas (Adriaenssens et al., 2015; Wen et al., 2016). To solve this problem, Brenninkmeijer and VanYperen (2003) tested different approaches and concluded that “exhaustion + 1” is the most recommended approach. This means that individuals are determined as burned out when having high levels of exhaustion and either high levels of cynicism or low levels of professional efficacy. This approach is in line with the idea that exhaustion is the core symptom of burnout, also the only dimension present in all different definitions and assessment tools for burnout. Therefore, exhaustion is a necessary symptom to set the “diagnosis” of burnout.

Due to the substantially various definitions and the impossibility to compare burnout prevalences across studies, we also directly compared the burnout scores between our results and studies using the same tool. Compared with the specific burnout scores of medical staff in different studies in China, this study found that the scores of medical staff on the EE and CY subscale were extremely higher, while the score on the PE subscale was lower (Wu et al., 2008, 2011, 2013, 2014; He et al., 2019). Taken together, it is speculated that there is a significant negative correlation between the long-term COVID-19 pandemic and the burnout experience of medical staff, although the criteria for the diagnosis of burnout are different.

Among the related factors of job burnout, job-related factors are the most concerned and discussed in detail under the burden of the COVID-19 pandemic. First of all, the occupation was closely related to job burnout. Compared with doctors and medical technicians, nurses are most likely to experience job burnout, which is consistent with many previous studies (Alacacioglu et al., 2009; Wu et al., 2011; Chou et al., 2014; Schooley et al., 2016) and the latest surveys conducted during the COVID-19 pandemic (Hu D. et al., 2020; Matsuo et al., 2020; Zhang Y. et al., 2020). Nursing job burnout has become a global phenomenon. In hospitals in the United States, there is a shortage of nursing staff, resulting in a high patient-to-nurse ratio, persistent emotional exhaustion, and job dissatisfaction (Aiken et al., 2002). A cross-sectional study of 12 European countries found that longer shifts (12 h or more) were associated with job burnout (Dall’Ora et al., 2015). The difference in working environment between countries limits the promotion of research in western countries. According to the few pieces of literature in China, the sense of professional efficacy of nurses is lower than that of doctors (Wu et al., 2013, 2014). For Chinese nurses, the large population base leads to a high nurse-patient ratio. Compared with doctors and medical technicians, nursing is a relatively low-paid profession in China. The reform of health care system policy and management strategy is accompanied by economic reform, which aggravates the great pressure and burnout of nurses (Wu et al., 2010). To make matters worse, in the early days of COVID-19 pandemic, medical personnel were not equipped with protective equipment and tested for coronavirus. As the main caregivers of patients, nurses have direct contact with infected patients many times a day when performing their duties. Therefore, compared with other medical staff, nurses face greater health risks, and consequently bear more psychological burden. Another explanation may be that nurses are mainly women, and they may bear more psychopathological burdens in outbreaks that threaten the health of family members or affect the care of children. Previous studies have demonstrated that women are more likely to suffer from Posttraumatic Stress Disorder (PTSD) and Posttraumatic Stress Symptoms (PTSS), and have more depressive and anxiety symptoms in the face of every coronavirus outbreak (Buselli et al., 2020; Carmassi et al., 2020).

The length of service was significantly correlated with every dimension of the burnout experience. Less than 20 years of service was risky for job burnout. The medical staff with a working life of 6–10 years had the strongest sense of emotional exhaustion and cynicism and the lowest sense of professional efficacy. Rich work experience after long service may contribute to higher esteem and better emotional regulation. It is reasonable that medical staff with work experience had enhanced psychological preparation and knowledge of infection control, and reduced the level of job burnout. The previous studies found that experienced nurses had a lower risk of violence in the workplace and a higher tolerance for patient aggression (Whittington, 2002; Edward et al., 2014). Young and inexperienced employees may be more nervous about highly contagious diseases.

Interestingly, we found that prolonged daily working hours were only associated with emotional exhaustion, not with cynicism and professional efficacy. As the relationship between excessive workload and a higher level of burnout has been well proved, many studies have proposed limiting working hours as the first step to prevent burnout (Gopal et al., 2005; Martini et al., 2006; Dugani et al., 2018). It should be noted that reducing working hours may not necessarily reduce cynicism and improve professional effectiveness. Moreover, during the COVID-19 epidemic, the huge number of infections and the exponential spread of coronavirus made the workload impossible to reduce. Therefore, during the pandemic, other more feasible methods are needed to alleviate the burnout experience.

Another important finding is that during the COVID-19 outbreak, job burnout of medical staff was positively correlated with depression. The latest report has demonstrated that during the COVID-19 outbreak, medical workers are twice as likely to suffer from depressive symptoms and other psychological disorders as the general population (Lu et al., 2020). The bi-directional link between burnout and depression has been widely recognized. Longitudinal studies have shown that an increase in burnout levels can predict a subsequent increase in depressive symptoms (Bianchi et al., 2015). As a result, the increase in depression is likely to be the result of exposure to unprecedented work-related stress during the COVID-19 pandemic, and vice versa. It is worth noting that due to the cross-sectional design, this study did not prove the causal relationship between burnout and depression.

This study has several limitations. First, this cross-sectional survey conducted at a single time point could not compare burnout levels before and after the outbreak. Moreover, there was relatively limited information on the specific factors of outbreaks that contribute to an increase in the prevalence of burnout. Therefore, our findings cannot reveal the causal relationship between the COVID-19 outbreak and high levels of job burnout. Second, as there is no consensus on the diagnosis of job burnout, it is difficult to directly compare the prevalence of job burnout. A recent review found that the existing literature used at least 47 different definitions of the prevalence of burnout when using the MBI tool to measure burnout (Rotenstein et al., 2018). Therefore, it is necessary in future studies to reach a consensus on how to classify different degrees of job burnout. Third, the levels of job burnout in the health care profession were not be compared with that of other occupations, as other industries were almost completely shut down during the pandemic. Therefore, the special impact of the COVID-19 epidemic on medical staff was not investigated. Fourth, there may be a sampling bias. The sample was composed of most female subjects who were more vulnerable to traumatic events. Hence, caution should be taken when extending our findings to other populations. Fifth, psychiatric evaluation of the samples was not performed before the study. As previously reported, pre-existing mental illness or susceptibility may have affected the development of burnout and depressive symptoms during the COVID-19 pandemic (Fiorillo et al., 2020). Sixth, the existence of burnout was investigated using an online self-administered questionnaire, which may compromise the reliability and validity of the measurement.

In summary, our report showed that there was a high rate of burnout among medical staff in China, which is likely to intensify during the COVID-19 pandemic. Occupation, length of service, working hours, and several individual variables, including age, sex, pre-existing physical diseases, and family income, are determinants of job burnout scores. The experience of burnout hinders the fight against the epidemic situation of COVID-19 and has a lasting negative impact on mental health. During and after the COVID-19 pandemic, intervention measures such as mindfulness-based decompression are urgent to deal with stress and solve the job burnout of medical staff. Psychological evaluation and psychological counseling should be carried out for medical staff on a long-term and regular basis. Our study suggests that when providing mental health services, more attention should be paid to young and less experienced medical staff, especially nurses. Compared with western countries, there are relatively few studies on job burnout of medical staff in China. Therefore, even after the COVID-19 pandemic, it is necessary to conduct more investigations on the causes and consequences of burnout and take effective intervention measures to prevent burnout.

## Data Availability Statement

The raw data supporting the conclusions of this article will be made available by the authors, without undue reservation, to any qualified researcher.

## Ethics Statement

The studies involving human participants were reviewed and approved by the Ethics Committee of the Institute of Psychology, Chinese Academy of Sciences. The patients/participants provided their written informed consent to participate in this study.

## Author Contributions

LH and YZ were responsible for statistical analysis and manuscript drafting. TL and XYZ were responsible for study design and writing review. YN, SL, ZL, and WQ were involved in statistical analysis and the manuscript revision. LZ, JY, and XZ were responsible for data acquirement. All the authors critically reviewed the manuscript and gave final approval for its publication.

## Conflict of Interest

The authors declare that the research was conducted in the absence of any commercial or financial relationships that could be construed as a potential conflict of interest.

## Footnotes

1 https://www.who.int/emergencies/diseases/novel-coronavirus-2019
